# How Do Nurses Navigate the Challenges of Risk Management? Results from a Cross-Sectional Survey in Southern Italy

**DOI:** 10.3390/healthcare14172871

**Published:** 2026-09-07

**Authors:** Domenico Ponticelli, Carmine Del Giudice, Davide Rozza, Elia Maglia, Lorenzo Losa, Ippazio Cosimo Antonazzo, Carla Amigoni, Marco Italo D’Orso, Anna Zampella, Domenico Tartaglia, Mara Noemi Fede, Fortuna Gallucci, Roberto Magliuolo, Antonella Arcari, Antonio Rainone, Giulio Geraci, Gaetano Mottola, Davide Ausili, Lorenzo Giovanni Mantovani, Pietro Ferrara

**Affiliations:** 1 Clinica Montevergine SpA, 83013 Mercogliano, Italy; 2 Centre for Public Health Research, University of Milan–Bicocca, 20900 Monza, Italy; 3 Department of Environmental and Prevention Sciences, University of Ferrara, 44121 Ferrara, Italy; 4 Nursing Service Directorate (SITR Lombardia), IRCCS Istituto Auxologico Italiano, 20149 Milan, Italy; 5 Independent Researcher, 81030 Lusciano, Italy; 6 Independent Researcher, 83100 Avellino, Italy; 7 Department of Medicine and Surgery, “Kore” University of Enna, 94100 Enna, Italy; 8 Unit of Internal Medicine, “Umberto I” Hospital, 94100 Enna, Italy; 9 School of Medicine and Surgery, University of Milano-Bicocca, 20900 Monza, Italy; 10 Laboratory of Public Health, IRCCS Istituto Auxologico Italiano, 20149 Milan, Italy

**Keywords:** clinical governance, incident reporting, nursing, patient safety, risk management, safety culture

## Abstract

**Background and Objectives**: Clinical risk management is essential to patient safety. Nurses are strategically positioned to identify unsafe conditions, near misses, and adverse events. This study assessed nurses’ knowledge of selected clinical risk management definitions, perceptions of patient safety, and self-reported experience of patient-safety event reporting in a cardiovascular hospital in Southern Italy. **Methods**: A cross-sectional anonymous survey was conducted among registered nurses using a structured questionnaire. Knowledge was measured through a 0–4 index based on correct answers regarding safety culture, near miss, sentinel event, and incident reporting. Perceived overall patient safety was analyzed as an ordinal outcome, and ever reporting of patient-safety events as a binary outcome. Multivariable regression models explored associated factors. **Results**: The study included 88 nurses; 75.0% were female, and the median age was 47 years (IQR 37–54). Overall, 77.3% reported previous updating on risk management. The median knowledge index was 3 (IQR 2–4), and 62.5% answered at least three of four items correctly. Patient-safety culture domains received high importance scores, with medians ranging from 9 to 10. Overall patient safety was rated as good, very good, or excellent by 63.6% of respondents, and 61.4% had reported at least one patient-safety event. Knowledge was associated with gender and years of professional experience. Higher perceived safety was associated with perceived technological adequacy and patient confidence in care. **Conclusions**: Beyond the setting-specific findings, the study illustrates how combining factual knowledge, perceived safety, reporting experience, and locally relevant process indicators may support organizational diagnosis and help identify areas requiring more focused quality-improvement assessment.

## 1. Introduction

Patient safety is a core dimension of healthcare quality and a central objective of clinical governance. Despite major advances in standards of care, technologies, and organizational procedures, preventable harm remains a persistent challenge for healthcare systems [1]. The World Health Organization has emphasized the need to reduce avoidable harm in healthcare and has identified patient safety as a strategic priority for health systems worldwide [2]. Within this framework, clinical risk management represents a structured approach to identifying, assessing, preventing, and learning from risks that may compromise the safety of patients and the quality of care [3].

Nurses play a crucial role in clinical risk management [4]. Because of their continuous presence at the bedside and their direct involvement in monitoring, administering care, coordinating activities, and communicating with patients and other professionals, they are often among the first to identify unsafe conditions, procedural deviations, communication failures, near misses, and adverse events [5,6,7]. Their ability to recognize and report risk-related situations is therefore essential for transforming individual episodes into opportunities for organizational learning and safety improvement [8]. However, effective risk management does not depend only on the availability of procedures or reporting systems. It also requires that nurses have adequate knowledge of key concepts, confidence in organizational processes, and operate within a positive safety culture [6,9].

Patient safety culture includes several interrelated domains, such as teamwork, staffing, handoffs and transitions, communication openness, management support, feedback and learning from events, frequency of event reporting, and non-punitive response to error [10,11]. These dimensions are closely connected to nursing work, which relies on continuous patient monitoring, coordination within and across units, accurate documentation, and effective communication during shift changes and care transitions [7,12,13]. In highly specialized settings, the complexity of care, the use of advanced technologies, and the need for timely multidisciplinary decisions further increase the importance of a robust safety culture [14,15].

Reporting behaviour should, however, be distinguished from factual knowledge of risk management terminology. The decision to report an adverse event or near miss may be influenced by the interaction of individual, behavioural, and organizational factors, including perceived blame or punitive consequences, managerial and peer support, clarity and accessibility of reporting procedures, feedback following a report, the perceived usefulness of reporting for organizational learning, and the broader safety climate [16]. In the Italian context, the recently validated Italian version of the Reporting of Clinical Adverse Events Scale (I-RoCAES) provides a complementary framework for characterizing these attitudinal dimensions and understanding how individual and organizational factors may shape adverse-event reporting [16]. These perspectives complement descriptive assessments of knowledge, perceptions, and reporting experience, allowing the different components of reporting practice to be considered within a broader patient-safety framework.

Organizational assessment of patient safety requires balancing standardized measurement with local actionability. Validated instruments and routine reporting data provide important but incomplete perspectives, which can be complemented by context-specific assessments incorporating local procedures, technologies, and reporting practices [16]. In this context, local assessments of nurses’ knowledge, perceptions, and reporting experience can help healthcare organizations understand how risk management principles are embedded in daily practice. Institutional procedures and aggregate indicators of adverse events may provide only a partial picture of patient safety, as they do not necessarily reflect how frontline professionals interpret risk, perceive the safety climate, or interact with reporting systems [17,18]. Understanding these frontline perspectives may help identify actionable gaps between formal risk-management systems and their implementation in everyday clinical practice, thereby informing targeted interventions to strengthen reporting, feedback, and organizational learning.

The present study aimed to assess clinical risk management among nurses working in a specialized cardiovascular hospital in Southern Italy. Specifically, the study evaluated nurses’ knowledge of key risk management concepts, their perceived level of patient safety within their unit or working area, and their previous experience with patient-safety event reporting. By examining these complementary dimensions, the study sought to identify factors associated with risk management preparedness and practice, and to provide evidence to support local patient-safety and clinical governance strategies.

## 2. Materials and Methods

### 2.1. Study Design

This cross-sectional survey was conducted in December 2024 in a cardiology hospital situated in southern Italy. This facility focuses on the diagnosis, treatment, and care of patients with cardiovascular diseases and heart-related conditions. It is equipped with highly specialized healthcare professionals, state-of-the-art diagnostic technologies, and tailored treatment options dedicated to cardiac care. The target population included registered nurses. Inclusion criteria were as follows: (1) current employment as a nurse, (2) at least one year of professional experience, and (3) direct involvement in clinical care. Nurses on extended leave or with administrative roles exclusively were excluded.

Given the single-hospital design of the study, all eligible nurses were invited to participate. Accordingly, the study was not based on a formal a priori sample size calculation and was not powered to detect small between-group differences. However, to contextualize the precision of the achieved sample size, we performed a post hoc precision assessment using data from a previous Italian survey on clinical risk management conducted among 98 nurses, in which 93% of participants were aware of the definition of risk management [19]. Assuming an expected proportion of 93% and a two-sided 95% confidence level, a sample of 88 nurses provides an expected confidence interval half-width of approximately 5.3 percentage points. Therefore, although the present sample size remains limited for multivariable modelling, it provides acceptable precision for estimating key descriptive proportions relevant to the study objectives.

### 2.2. Survey Instrument

Data were collected using a structured, self-administered questionnaire developed to assess nurses’ knowledge, perceptions, and practices regarding clinical risk management. The questionnaire was designed by the study team in collaboration with experts in public health, nursing, and risk management, taking into account the main domains of patient safety culture and incident reporting, as well as previous experience reported in the literature [19,20].

The questionnaire was developed for the specific descriptive and exploratory objectives of the study. The use of an ad hoc instrument allowed the inclusion of factual and context-specific items concerning local procedures, technologies, and reporting practices.

The questionnaire, included as Supplementary Materials, consisted of four sections. Section A collected demographic and professional information, including gender, year of birth, highest educational qualification, years of professional experience, and previous exposure to training or educational content on clinical risk management. Section B explored knowledge, opinions and perceptions regarding the culture and tools for clinical risk management in the hospital. Knowledge-based items included definition of safety culture, definition of near miss, definition of sentinel event, and definition of incident reporting. This section included a multiple-choice item on the meaning of safety culture; a series of 1-to-10 rating-scale items assessing the perceived importance of patient-safety domains, including teamwork, inter-unit collaboration, staffing, handoffs and transitions, managerial support, organizational measures for preventing adverse events, procedures, continuous learning, open communication, non-punitive response to errors, leadership support, overall safety perception, and event-reporting frequency. Additional items covered the knowledge of company procedures for reporting adverse events and near misses, perceived adequacy of risk management training, and adequacy of technologies for reducing clinical risk. Section C assessed the perceived overall level of patient safety in the respondent’s unit or working area and the perceived safety of care from the patients’ perspective. Section D collected information on previous reporting experience and knowledge of key risk management concepts.

The survey used a combination of multiple-choice questions, ordinal response categories, Likert-type agreement scales, frequency scales, and numerical rating scales. The questionnaire was anonymous; no personal identifiers or patient-level clinical data were collected, and responses were analyzed only in aggregate form. Participation was voluntary, and completion of the questionnaire was considered consent to use the data for research purposes.

An electronic survey platform specifically developed by the Information Technology unit of the hospital was used for data collection. Recruitment was conducted through direct email invitations sent to all eligible nurses. Before survey launch, the questionnaire was pre-tested with a convenience sample of 10 nurses representative of the target population. Feedback from this pilot phase led to minor refinements in wording and formatting to improve clarity and usability. These procedures were intended to support the content relevance, comprehensibility, and usability of the questionnaire. Internal consistency was assessed using Cronbach’s alpha for the set of 13 items evaluating the perceived importance of patient-safety culture domains. These items covered teamwork within units, collaboration across units, staffing adequacy, handoffs and task transitions, promotion of safety by supervisors/managers, organizational measures for the prevention of adverse events, company procedures, learning and continuous improvement, open communication regarding adverse events, non-punitive response to errors, management support, overall perception of safety, and frequency of event reporting. The 13-item set yielded a Cronbach’s alpha of 0.98; the implications are discussed in the Section 4.2.

### 2.3. Statistical Analysis

Descriptive statistics (frequencies, medians and interquartile ranges [IQR]) were used to summarize participant characteristics and survey responses, given the non-normal distribution of the quantitative variables. Three outcomes were defined a priori to capture distinct dimensions of risk management: knowledge, perceived safety, and reporting behaviour. The first outcome was familiarity with selected clinical risk management definitions, operationalized as a prespecified four-item additive knowledge index ranging from 0 to 4. One point was assigned for each correct answer to four knowledge-based items: definition of safety culture, definition of near miss, definition of sentinel event, and definition of incident reporting. The index was intended to represent the number of selected definitions correctly identified and the resulting score represented the number of correct answers provided by each respondent. Since the outcome was defined as the number of correct answers across four items, each contributing independently one point to a score ranging from 0 to 4, associations between participant characteristics and knowledge were assessed using a binomial regression model with a logit link. Results were expressed as odds ratios (ORs) and 95% confidence intervals (95% CIs), representing the association between each predictor and the odds of correctly answering the individual items included in the four-item knowledge index. The second outcome was the perceived level of overall patient safety in the respondent’s unit or working area, assessed through a specific questionnaire item with five ordered response categories: poor, sufficient, good, very good, and excellent. This variable was derived from the item asking respondents to rate the overall level of patient safety as poor, sufficient, good, very good, or excellent. Given the ordered nature of the response categories, this outcome was analyzed using ordinal logistic regression. Results were reported as coefficients and 95% CIs. The third outcome was ever reporting of patient-safety events during the professional career. This variable was derived from the item asking respondents how many patient-safety events they had reported during their professional career. Responses were dichotomized as no reported events versus at least one reported event. This binary outcome was analyzed using logistic regression, with results expressed as ORs and 95% CIs. The three regression models corresponded to three distinct outcomes defined a priori and were selected according to the measurement scale and distribution of each outcome.

Inferential analyses were performed in two stages, following the model-building approach recommended by Hosmer et al. [21]. Initially, univariable analyses were conducted to examine the association between each independent variable and the three outcomes of interest. For the three models, candidate independent variables included demographic and professional characteristics (gender, age, educational level, years of professional experience, and previous professional updating on risk management) and selected organizational and training-related variables (perceived adequacy of risk-management training, knowledge of organizational procedures for reporting adverse events and near misses, participation in organizational initiatives on incident reporting, perceived adequacy of technologies to reduce clinical risk, and nurses’ perception that patients felt care to be safe or unsafe). In addition, each of the three prespecified outcome domains was considered as a potential explanatory variable in models for the other outcomes, where conceptually appropriate. Variables with a *p*-value equal to or lower than 0.25 in univariable analysis were considered for potential inclusion in the multivariable regression models. Subsequently, according to a stepwise model-building strategy, variables with a *p*-value < 0.25 in multivariable analysis were retained for evaluation in the final models [21,22]. Final models included variables selected on the basis of statistical criteria, avoiding overfitting in relation to the available sample size. Variables retained in each final model are reported with the corresponding results. Statistical significance was set at a two-sided *p*-value < 0.05. Quantitative data were analyzed using Stata version 19.5 statistical software [23].

## 3. Results

Of the 107 eligible nurses invited to participate, 88 completed the survey (Table 1), yielding a response rate of 82.2%. Most respondents were female (n = 66, 75.0%), while 22 were male (25.0%). The median age was 47 years (IQR 37–54). Regarding educational level, 77 nurses (87.5%) had a bachelor’s degree, whereas 11 (12.5%) had a master’s degree or postgraduate education. Overall, 68 respondents (77.3%) reported previous professional updating on clinical risk management, while 20 (22.7%) did not.

The four-item knowledge index of selected clinical risk management concepts ranged from 0 to 4 correct answers. Overall, two nurses (2.3%) scored 0, 15 (17.1%) scored 1, 16 (18.2%) scored 2, 25 (28.4%) scored 3, and 30 (34.1%) scored 4. The median four-item knowledge index was 3 (IQR 2 to 4). Overall, 55 nurses (62.5%) answered at least three out of four knowledge items correctly, while 33 (37.5%) had a score of 0 to 2. When the individual components of the knowledge index were examined, the highest proportion of correct answers was observed for the definition of incident reporting, correctly identified by 67 nurses (76.1%). Correct answers were also frequent for the definition of safety culture (n = 61, 69.3%), sentinel event (n = 58, 65.9%), and near miss (n = 56, 63.6%).

Overall, 82.0% of respondents reported knowing the specific organizational procedures for reporting adverse events and near misses. Regarding the perceived adequacy of clinical risk management training to address the challenges of their profession, approximately three-quarters of respondents rated it as adequate, whereas the remaining respondents considered it inadequate or only poorly adequate. Similarly, the technologies used in the working area were perceived as adequate or highly adequate to reduce clinical risk by 77.3% of nurses. Complete results for these items are presented in Table S1 (Supplementary Materials).

Nurses assigned high importance to all patient-safety culture domains assessed on the 1–10 scale (Table 2).

Median scores ranged from 9 to 10 across all domains. The highest median scores were observed for handoffs and task transitions and for organizational measures for the prevention of adverse events, both with a median score of 10 (IQR 8 to 10). Most other domains, including teamwork within units, collaboration across units, staffing adequacy, managerial promotion of safety, company procedures, continuous learning, open communication, non-punitive response to errors, management support, overall perception of safety, and frequency of event reporting, had median scores of 9. The widest variability was observed for non-punitive response to errors (IQR 6.5 to 10) and frequency of event reporting (IQR 6 to 10).

The perceived overall level of patient safety in the respondent’s unit or working area was most frequently rated as good. Specifically, seven nurses (8.0%) rated patient safety as poor, 25 (28.4%) as sufficient, 33 (37.5%) as good, 19 (21.6%) as very good, and four (4.6%) as excellent. Regarding nurses’ perceptions of patients’ perceived safety of care, only 8 respondents (9.1%) answered that patients perceived care services as not very safe, whereas the remaining respondents reported that patients perceived care as safe or very safe.

**Figure 1:**
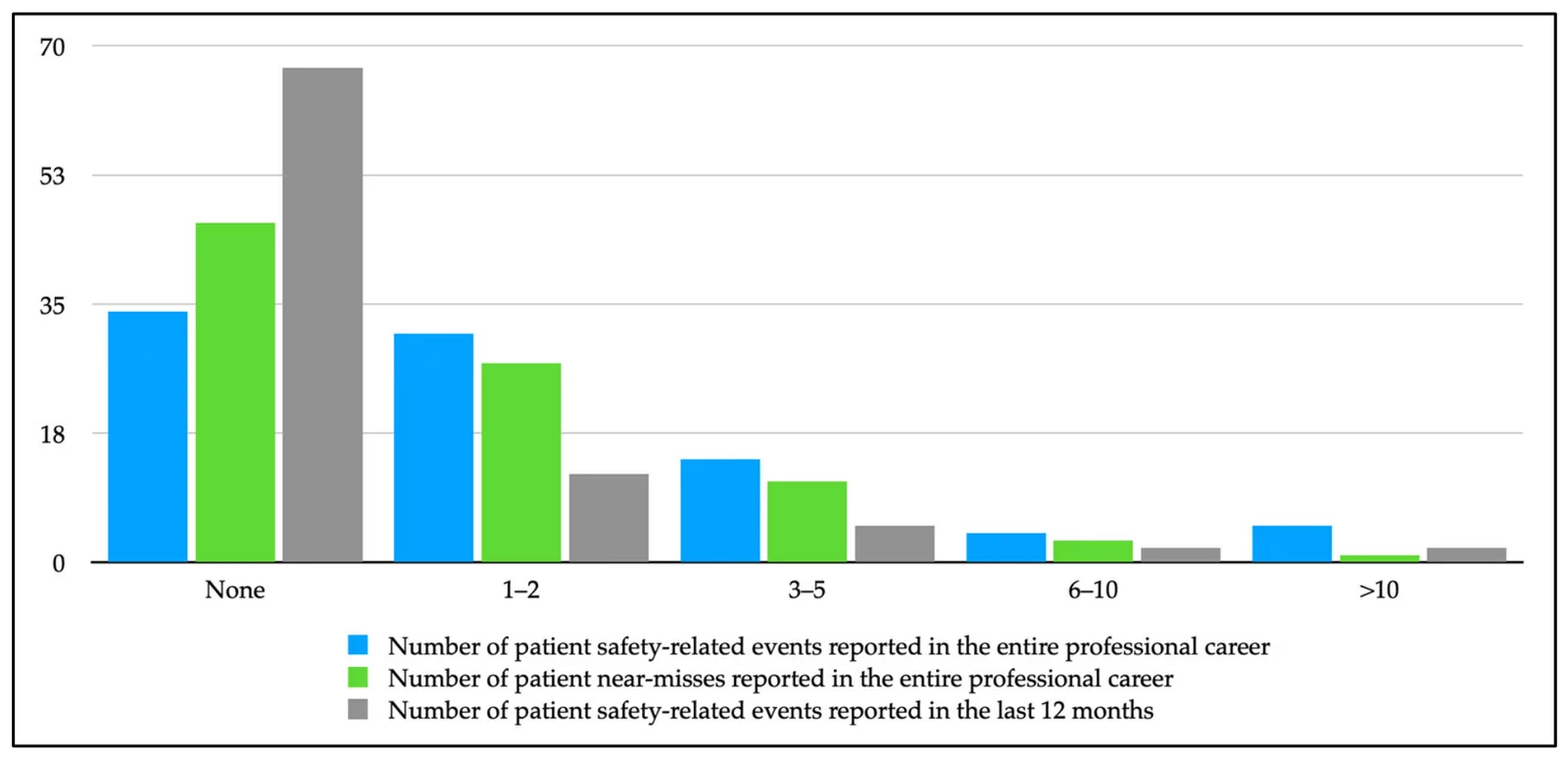
Distribution of patient-safety event and near-miss reporting among nurses.

Regarding previous reporting behaviour, 34 nurses (38.6%) reported that they had never reported a patient-safety event during their professional career. Among those who had reported at least one event, 31 (35.2%) reported 1–2 events, 14 (15.9%) reported 3–5 events, 4 (4.6%) reported 6–10 events, and 5 (5.7%) reported more than 10 events. Overall, 54 nurses (61.4%) had reported at least one patient-safety event during their professional career. As shown in Figure 1, the distribution of event and near-miss reporting was skewed toward lower reporting categories, with no reporting being the most frequent category for several reporting indicators, particularly for events reported in the previous 12 months.

In the binomial regression model (Table 3, Model 1), women had higher odds of correctly answering the four-item knowledge index (OR 2.19, 95% CI 1.21 to 3.98). A positive association was also seen for perceived adequacy of technologies to reduce clinical risk (OR 1.28, 95% CI 1.02 to 1.59). Years of professional experience were inversely associated with knowledge index, with each additional year of experience associated with lower odds of correctly answering the knowledge index (OR 0.96, 95% CI 0.94 to 0.99).

In the ordinal logistic regression model for perceived overall patient safety (Table 3, Model 2), perceived adequacy of technologies to reduce clinical risk was positively associated with higher perceived safety of technologies (coefficient 1.35, 95% CI 0.77 to 1.93). Nurses’ perception that patients felt care to be safe was also strongly associated with better perceived overall patient-safety level (coefficient 2.69, 95% CI 1.01 to 4.37). Conversely, having ever reported a patient-safety event was negatively associated with perceived overall patient safety (coefficient −0.82, 95% CI −1.61 to −0.02).

In the logistic regression model for ever reporting of patient-safety events (Table 3, Model 3), no predictor reached conventional statistical significance. Years of professional experience showed a positive association with ever reporting, although this did not reach statistical significance (*p* = 0.08). Nurses with a master’s degree or postgraduate education had higher odds of having reported at least one patient-safety event compared with those with a bachelor’s degree, but the association was imprecise and not statistically significant (*p* = 0.16).

## 4. Discussion

This cross-sectional survey provides updated insight into nurses’ knowledge, perceptions, and reporting experience regarding clinical risk management in a specialized cardiovascular hospital in Southern Italy. In doing so, it builds on earlier Italian surveys, which had already highlighted the relevance of knowledge, perceived severity of sentinel events, error reporting, and the use of reporting systems in clinical risk management [19,20]. Our evidence also confirmed the relevance of educational exposure, risk perception, and reporting behaviours in this field [20].

This study should primarily be interpreted as a setting-specific organizational assessment rather than as an attempt to estimate a generalizable level of clinical risk-management competence or safety culture. Its broader contribution lies in showing what can be learned when different sources of frontline information—factual knowledge, perceived safety, reporting experience, and perceptions of locally relevant organizational resources—are considered together. In our setting, these dimensions did not provide an entirely uniform picture: nurses attributed very high importance to patient-safety domains, while variability remained in knowledge of selected risk-management concepts and recent reporting activity was limited. This illustrates an important organizational diagnostic principle: apparently favourable findings in one dimension of patient safety do not necessarily imply equivalent performance in others, and reliance on a single indicator may therefore obscure areas requiring further assessment.

Risk management requires a shared understanding of key patient-safety concepts, including safety culture, near miss, sentinel event, and incident reporting [9,24]. For nurses, this knowledge provides a common language for recognizing, communicating, and managing risk-related situations in daily clinical practice [10]. In the present study, most respondents correctly identified the key concepts included in the four-item knowledge index, particularly incident reporting, followed by safety culture, sentinel event, and near miss. This finding suggests that the core terminology of clinical risk management is broadly familiar among the surveyed nurses. However, the variability across individual items, and the fact that approximately one third of respondents did not reach the highest levels of the knowledge index, indicate that there remains room for strengthening shared conceptual understanding [17,20]. Variability in knowledge of risk management terminology may reflect differences in training exposure, procedural familiarity, and institutional communication [25].

In the multivariable model, years of professional experience were inversely associated with the knowledge index; however, this finding should be interpreted cautiously. Indeed, it may reflect generational differences in exposure to formal patient-safety curricula, more recent integration of risk management concepts into professional training, or greater familiarity among younger professionals with standardized terminology related to incident reporting and safety culture [26,27]. It does not imply that more experienced nurses are less competent in clinical practice; rather, it suggests that continuous professional development should ensure that all staff, regardless of seniority, share the same updated language and operational definitions of risk management [26].

Incident reporting systems are a central tool for patient safety improvement, as they allow organizations to document adverse events, capture near misses, and identify unsafe conditions before they result in harm [28]. This function is particularly relevant for nurses, who provide continuous bedside care, monitor patients across different phases of the care pathway, and are often strategically positioned to recognize deviations from expected clinical processes [6,11,29]. In this study, a majority of nurses reported having submitted at least one patient-safety event during their professional career. Nevertheless, the distribution of reporting was skewed toward lower categories, with no reporting being common, particularly for events reported in the previous 12 months. This pattern is consistent with the well-known challenge of underreporting in patient-safety systems and reinforces the importance of making reporting procedures clear, accessible, useful, and visibly connected to learning and improvement [20,30,31,32].

The reporting measures used in this study should be interpreted as indicators of previous self-reported reporting experience rather than as direct measures of attitudes toward reporting or organizational reporting culture. They do not capture behavioural and organizational determinants such as perceived blame, expected organizational response, peer and managerial support, psychological safety, or the perceived usefulness of reporting. Within the scope of this study, these measures were used as part of an exploratory pilot assessment to examine whether reporting experience, factual knowledge, and perceived safety could be considered concurrently and whether meaningful relationships among these dimensions could be identified. Accordingly, the present findings should be regarded as a proof of concept rather than as validation of an organizational diagnostic or quality-improvement tool. Future studies should combine self-reported reporting experience with validated psychometric measures, such as the I-RoCAES [16], institutional reporting data, and formal assessments of acceptability and feasibility, in order to determine whether this type of multidimensional assessment can support routine quality-assurance or quality-improvement.

The absence of statistically significant predictors in the model for previous reporting experience suggests that the decision to report may be influenced by factors not fully captured by individual demographic or educational variables. Although years of professional experience and higher educational level showed positive directions of association, estimates were imprecise. Reporting is likely shaped by local culture, perceived psychological safety, previous feedback after reports, managerial responses, workload, and perceived usefulness of the reporting system [30]. Therefore, interventions aimed at increasing reporting should not rely exclusively on educational initiatives, but should also strengthen feedback loops, ensure clear and standardized procedures, enhance transparency regarding actions taken after reports, and promote a non-punitive approach to errors and near misses. From a behavioural and organizational perspective, even limited gaps in procedural knowledge or reporting practice are relevant, because when healthcare professionals are not consistently supported in applying correct procedures, informal or incorrect behaviours may become normalized and gradually embedded into routine practice [33].

The perceived overall level of patient safety was most frequently rated as good, while fewer respondents rated it as very good or excellent. This suggests that nurses generally recognized an acceptable safety environment, while still identifying potential margins for improvement [5]. The strong positive association between perceived adequacy of technologies and higher perceived safety is particularly relevant in a specialized cardiovascular hospital. In this context, technologies should be understood broadly, including also routine monitoring systems, imaging facilities, electronic documentation tools, alarm systems, infusion devices, devices used in operating rooms and intensive care units, and information systems supporting communication and traceability of clinical processes. In cardiovascular care, where patients often require continuous monitoring, invasive procedures, rapid decision-making, and coordinated transitions across units, the perceived adequacy and reliability of these technologies may directly contribute to nurses’ sense of a safer care environment. Technology may therefore be perceived not simply as a clinical resource, but as part of the organizational infrastructure that supports early detection of clinical deterioration, standardization of procedures, reduction in preventable errors, and timely response to risk [34].

Nurses’ perception that patients felt care to be safe was also strongly associated with better perceived overall patient safety. This finding should be interpreted as a perceptual alignment between nurses’ assessment of the safety climate and their view of patients’ experience of care. Although patients were not directly surveyed, nurses’ perceptions of patients’ confidence in care may reflect broader relational and organizational dimensions, including communication, trust, continuity, and perceived reliability of care processes [35,36]. Future studies could expand this perspective by directly integrating patient-reported experience measures related to safety.

Conversely, having ever reported a patient-safety event was negatively associated with perceived overall patient safety. This finding may appear counterintuitive, but it is plausible. Nurses who have reported events may be more aware of safety vulnerabilities, more exposed to adverse or near-miss situations, or more likely to work in areas where clinical complexity increases the visibility of risk. Alternatively, professionals working in units perceived as less safe may be more likely to encounter and report patient-safety events. Because of the cross-sectional design, the direction of this association cannot be established. Rather than suggesting that reporting reduces perceived safety, this result highlights the close relationship between reporting behaviour, risk awareness, and safety climate [10,11,37].

### 4.1. Implications for Organizational Assessment and Quality Improvement

Three lessons from this assessment may be transferable to other organizations. First, organizational diagnosis should distinguish between knowledge, perceptions, and interaction with safety processes rather than treating them as manifestations of a single construct [38]. Second, context-specific questions may improve the actionability of an assessment by linking responses to local procedures, technologies, and reporting pathways, while complementing validated instruments when standardized measurement or benchmarking is required. Third, survey findings should be regarded as signals for further investigation and triangulated with incident-reporting data, audits, qualitative information, and other quality indicators. Thus, the transferable element of this study lies not in the numerical estimates observed in this hospital, but in the diagnostic logic used to integrate different sources of frontline information and identify priorities for further assessment and quality improvement.

Consistent with this approach, risk management should be viewed as a multidimensional process in which knowledge, reporting experience, and perceived safety provide complementary information. Educational initiatives should therefore be embedded within broader clinical governance strategies that include clear and accessible reporting procedures, managerial support, feedback after reporting, and visible organizational learning [9,25]. In the present setting, the findings may help identify areas warranting further local assessment, including refresher training and reporting processes. However, any organizational intervention should be informed by additional evidence and, where possible, evaluated using validated instruments, institutional reporting data, audits, and prospective assessment.

### 4.2. Limitations

This study has several limitations. First, its cross-sectional design precludes causal inference, and the observed associations should therefore be interpreted as exploratory and hypothesis-generating. Second, the study was conducted in a single specialized cardiovascular hospital in Southern Italy and included fewer than 100 participants, limiting statistical power, the precision of some estimates, and the generalizability of the findings to other healthcare settings, regions, or professional populations. Although all eligible nurses were invited and the response rate was relatively high, participation was voluntary and some degree of participation bias cannot be excluded, as respondents may have differed from non-respondents in their interest in or familiarity with clinical risk-management issues. Multivariable models were therefore kept parsimonious and should be interpreted with caution. Third, although expert review and pre-testing supported the clarity, contextual relevance, and usability of the questionnaire, the instrument did not undergo formal psychometric validation. The study-specific measures should therefore be interpreted as exploratory operational indicators rather than as validated measures of comprehensive clinical risk-management competence or organizational safety culture. Fourth, all variables were collected contemporaneously from the same respondents using the same self-administered questionnaire, introducing the possibility of common-method bias. Some observed associations, particularly those involving perceptual measures, may therefore partly reflect shared response tendencies, consistency effects, recall, or social desirability rather than relationships between fully independent underlying constructs. Reporting experience was also based on respondents’ recollection rather than on objective institutional incident-reporting records. Fifth, the four-item knowledge index assessed familiarity with selected clinical risk management concepts but could not capture the broader behavioural, organizational, procedural, and professional competencies involved in patient-safety practice. Similarly, perceived patient safety and patient confidence were reported from the nurses’ perspective and should not be considered direct measures of patient-reported safety or experience. In addition, the very high Cronbach’s alpha observed for the 13 items assessing the perceived importance of patient-safety domains (α = 0.98) should be interpreted cautiously. The marked concentration of responses at the upper end of the scale may have contributed to this high internal-consistency estimate by reducing response variability. Further work is therefore needed to refine the measurement of the perceived importance of patient-safety domains.

Despite these limitations, the study provides useful local evidence on how nurses understand, perceive, and engage with clinical risk management in a highly specialized hospital setting. Importantly, these limitations primarily constrain the external generalizability of the numerical estimates and observed associations, rather than the potential transferability of the organizational diagnostic approach. Other healthcare organizations could apply a similar multidimensional framework while adapting individual items to their own procedures and combining context-specific assessments with validated instruments and objective safety data [16]. Whether this approach provides added value across different organizational settings should be evaluated prospectively.

## 5. Conclusions

This study identified variability in nurses’ knowledge of selected risk-management concepts, perceived safety, and reporting experience within a specialized cardiovascular hospital. At the organizational level, these findings provide signals for further assessment of training, reporting pathways, feedback mechanisms, and non-punitive learning processes. More broadly, the study illustrates the value of considering knowledge, perceptions, reporting experience, and context-specific organizational factors as complementary rather than interchangeable sources of information. The numerical findings remain setting-specific, but this multidimensional diagnostic logic may be useful to other healthcare organizations seeking to identify locally relevant patient-safety priorities. Such assessments should complement, rather than replace, validated safety-culture instruments, routine incident-reporting data, and other quality indicators.

## Figures and Tables

**Table 1:** Selected characteristics of the study population (N = 88).

Characteristic	N	Percentage
Gender		
Female	66	75.0
Male	22	25.0
Age (median and interquartile range)	47 (37–54)	
Educational level		
Bachelor’s degree	77	87.5
Master’s degree or postgraduate education	11	12.5
Years of professional experience (median and interquartile range)	18 (8.5–28)	
Primary working unit		
Cardiology	8	9.1
Interventional cardiology	9	10.2
Electrophysiology	9	10.2
Cardiac surgery	12	13.6
Post-surgery intensive care	14	15.9
Coronary care	15	17.1
Cardio-pulmonology	11	12.5
Outpatient cardiology unit	9	10.2
Radiology service	1	1.1
Previous professional updating on risk management		
Yes	68	77.3
No	20	22.7

**Table 2:** Perceived importance of patient-safety domains among nurses.

Patient-Safety Domain	Median	IQR
Teamwork within units	9	8–10
Collaboration across units	9	7.5–10
Staffing adequacy within units	9	7–10
Handoffs and task transitions	10	8–10
Promotion of safety by supervisors/managers	9	8–10
Organizational measures for the prevention of adverse events	10	8–10
Company procedures	9	7.5–10
Actions and initiatives for learning and continuous improvement	9	7–10
Open communication regarding adverse events	9	7–10
Non-punitive response to errors	9	6.5–10
Management support for patient safety	9	7–10
Overall perception of safety	9	7.5–10
Frequency of event reporting	9	6–10

Note: Items were rated on a 1–10 numerical rating scale, with higher scores indicating greater perceived importance. IQR, interquartile range.

**Table 3:** Multivariable regression models for risk management knowledge, reporting behaviour, and perceived patient safety among nurses.

Model 1. Four-item knowledge index of selected clinical risk management concepts	Predictor	OR	95% CI	*p*-value
	Gender (Reference category: Men)	2.19	1.21 to 3.98	0.01
	Educational level: master’s degree/postgraduate vs. bachelor’s degree	0.54	0.23 to 1.28	0.16
	Years of professional experience	0.96	0.94 to 0.99	0.01
	Perceived adequacy of technologies to reduce clinical risk	1.28	1.02 to 1.59	0.03
Model 2. Perceived overall patient safety	Predictor	Coefficient	95% CI	*p*-value
	Perceived adequacy of technologies to reduce clinical risk	1.35	0.77 to 1.93	<0.001
	Nurses’ perception that patients felt care was safe (vs. unsafe)	2.69	1.01 to 4.37	0.002
	Ever reporting of patient-safety events	−0.82	−1.61 to −0.02	0.04
Model 3. Ever reporting of patient-safety events	Predictor	OR	95% CI	*p*-value
	Educational level: master’s degree/postgraduate vs. bachelor’s degree	3.16	0.63 to 15.95	0.16
	Years of professional experience	1.04	1.00 to 1.08	0.08

Abbreviations: OR, odds ratio; 95% CI, 95% confidence interval. Notes: Model 1 was a binomial regression model with a logit link for the composite four-item knowledge index, defined as the number of correct answers out of four selected risk management knowledge items. Model 2 was an ordinal logistic regression model for perceived overall patient safety. Model 3 was a logistic regression model for having reported at least one patient-safety event during the professional career.

## Data Availability

Data and supporting materials associated with this study will be provided upon request by contacting the corresponding author.

## Supplementary Materials

The following supporting information can be downloaded at https://www.mdpi.com/article/10.3390/healthcare14172871/s1. Study questionnaire; Table S1. Distribution and regression coding of perceived adequacy of risk management training and technologies used to reduce clinical risk.
