# Supplementary material for: How Do Nurses Navigate the Challenges of Risk Management? Results from a Cross-Sectional Survey in Southern Italy
Source: Healthcare (Basel). 2026 Sep 7;14(17):2871. doi: 10.3390/healthcare14172871 (PMC13566045; doi:10.3390/healthcare14172871)
Supplement: Supplementary file 1 [file healthcare-14-02871-s001.zip › healthcare-4480507-supplementary.pdf]

Article

How do nurses navigate the challenges of risk management? Results from a cross-sectional survey in Southern Italy

Supplementary material

–

Appendix to Methods

Study questionnaire

This appendix includes the English-language version of the structured, self-administered questionnaire developed specifically for this study.

A. DEMOGRAPHIC AND PROFESSIONAL INFORMATION

A1. Gender:

☐ M ☐ F

A2. Year of birth: __________

A3. What is the highest educational qualification you have obtained?

☐ Bachelor's degree
☐ Master's degree
☐ Postgraduate master's course

A4. For how many years have you been practicing this profession? (number of years) __________

A5. In which unit/department do you work?

☐ Cardiology
☐ Electrophysiology
☐ Cardiac surgery
☐ Cardiopneumology
☐ Post-surgery intensive care unit
☐ Coronary care unit
☐ Interventional cardiology operating room
☐ Cardiac surgery operating room
☐ Outpatient unit
☐ Radiology service

A6. How do you usually update your professional knowledge?

☐ CME courses
☐ Scientific society congresses
☐ Monothematic conferences
☐ Searching for guidelines
☐ Scientific journals
☐ Internet/media
☐ Other: ____________________
☐ None

A7. Have you ever studied concepts related to clinical risk management through any of the updating sources listed in question A6?

☐ Yes ☐ No

SECTION B

In this section, you will be asked for knowledge and opinions regarding the culture and tools for clinical risk management in the hospital.

B1. What does "safety culture" mean?

☐ Adopting rigid protocols without exceptions.

☐ Ensuring that errors are avoided at all costs.

☐ Promoting an environment in which healthcare professionals can report errors without fear of sanctions.

☐ Reducing healthcare costs through error prevention.

B2. What is a near miss?

☐ An adverse event of particular severity, potentially preventable, that may result in death or serious harm to the patient and that causes citizens to lose trust in the healthcare service.

☐ Any unexpected event related to the care process.

☐ An error with the potential to cause an adverse event that does not occur by chance, because it is intercepted, or because it has no adverse consequences for the patient.

☐ A method for reporting adverse events.

B3. What is a sentinel event?

☐ An adverse event of particular severity, potentially preventable, that may result in death or serious harm to the patient and that causes citizens to lose trust in the healthcare service.

☐ Any unexpected event related to the care process.

☐ An error with the potential to cause an adverse event that does not occur by chance, because it is intercepted, or because it has no adverse consequences for the patient.

☐ The first event of a given type that occurs in a ward/unit.

B4. What is incident reporting?

☐ A tool used exclusively to monitor the costs associated with errors in healthcare.

☐ A mandatory method for documenting all incidents only for legal purposes.

☐ A tool for reporting, analyzing, and preventing adverse events and near misses.

☐ A system that may include only reports intended to identify the person responsible for errors.

B5. Do you know the specific organizational procedures for reporting adverse events and near misses?

☐ Yes

☐ No

B6. On a scale from 1 to 10, please indicate how important you consider the following aspects for patient safety (1 indicates no importance and 10 indicates high importance):

Aspect	Score									
Teamwork within operating units	1	2	3	4	5	6	7	8	9	10
Collaboration among different operating units	1	2	3	4	5	6	7	8	9	10
Staffing level and mix within operating units	1	2	3	4	5	6	7	8	9	10
Handoffs and task transitions	1	2	3	4	5	6	7	8	9	10
Promotion of safety by supervisors/managers	1	2	3	4	5	6	7	8	9	10

Organizational measures for the prevention of adverse events	1	2	3	4	5	6	7	8	9	10
Organizational procedures	1	2	3	4	5	6	7	8	9	10
Actions and initiatives for learning and continuous improvement	1	2	3	4	5	6	7	8	9	10
Open communication regarding adverse events	1	2	3	4	5	6	7	8	9	10
Non-punitive response to errors	1	2	3	4	5	6	7	8	9	10
Management support for patient safety	1	2	3	4	5	6	7	8	9	10
Overall perception of safety	1	2	3	4	5	6	7	8	9	10
Frequency of event reporting	1	2	3	4	5	6	7	8	9	10

B7. Do you think that training on clinical risk management is adequate to address the challenges of your profession?

☐ No.

☐ Slightly adequate.

☐ Adequate.

☐ Quite adequate.

☐ Fully adequate.

B8. Do you think that the technologies used in your work area are adequate to reduce clinical risk?

☐ No.

☐ Slightly adequate.

☐ Adequate.

☐ Quite adequate.

☐ Fully adequate.

SECTION C

In this section, you will be asked for general information regarding clinical risk management in your professional practice.

C1. How would you rate the overall level of patient safety in the unit/area where you work?

☐ Poor
☐ Sufficient
☐ Good
☐ Very good
☐ Excellent

C2. In the unit/area where you work, patients perceive care services as:

☐ Not safe at all
☐ Not very safe
☐ Safe
☐ Very safe
☐ I don't know

SECTION D

In this section, you will be asked to answer general questions regarding clinical risk management in your professional career.

D1. During your professional career, how many patient-safety-related events have you reported?

☐ None
☐ Between 1 and 2
☐ Between 3 and 5
☐ Between 6 and 10
☐ More than 10

D2. If you have reported events, how many did you report even though they had not caused harm to the patient?

☐ None
☐ Between 1 and 2
☐ Between 3 and 5
☐ Between 6 and 10
☐ More than 10

D3. In the last 12 months, how many patient-safety-related events have you reported?

☐ None
☐ Between 1 and 2
☐ Between 3 and 5
☐ Between 6 and 10
☐ More than 10

Appendix to Results

Table S1. **Distribution and regression coding of perceived adequacy of risk management training and technologies used to reduce clinical risk**

	Items		
	Do you think that training on clinical risk management is adequate to address the challenges of your profession?	Do you think that the technologies used in your work area are adequate to reduce clinical risk?	
	N (%)	N (%)	Ordinal coding used in regression models
No	12 (14.8)	19 (21.6)	-2
Slightly adequate	2 (2.3)	1 (1.1)	-1
Adequate	31 (35.2)	39 (44.3)	0
Quite adequate	30 (34.1)	23 (26.1)	+1
Fully adequate	12 (13.6)	6 (6.8)	+2
